# Flocculated meltwater particles control Arctic land-sea fluxes of labile iron

**DOI:** 10.1038/srep24033

**Published:** 2016-04-06

**Authors:** Thor Nygaard Markussen, Bo Elberling, Christian Winter, Thorbjørn Joest Andersen

**Affiliations:** 1 Center for Permafrost (CENPERM), Department of Geosciences and Natural Resource Management, University of Copenhagen, Øster Voldgade 10, DK-1350 Copenhagen, Denmark; 2 Center for Marine Environmental Sciences (MARUM), University of Bremen, Leobener Str., D-28359 Bremen, Germany

## Abstract

Glacial meltwater systems supply the Arctic coastal ocean with large volumes of sediment and potentially bioavailable forms of iron, nitrogen and carbon. The particulate fraction of this supply is significant but estuarine losses have been thought to limit the iron supply from land. Here, our results reveal how flocculation (particle aggregation) involving labile iron may increase horizontal transport rather than enhance deposition close to the source. This is shown by combining field observations in Disko Fjord, West Greenland, and laboratory experiments. Our data show how labile iron affects floc sizes, shapes and densities and consequently yields low settling velocities and extended sediment plumes. We highlight the importance of understanding the flocculation mechanisms when examining fluxes of meltwater transported iron in polar regions today and in the future, and we underline the influence of terrestrial hotspots on the nutrient and solute cycles in Arctic coastal waters.

Recent studies have shown the large supply of labile and potentially bioavailable forms of iron (Fe)^1^,^2^,^3^,^4^,^5^,^6^,^7^, nitrogen and carbon^8^,^9^ from glacial meltwater systems to the Arctic coastal ocean. Labile Fe, which is defined based on the method of extraction, as well as, e.g., carbon, nitrogen and aluminium, is bound to particles^1^,^6^,^7^,^10^, making sediment the main transport agent of Fe and other elements to estuarine waters^1^,^11^,^12^,^13^. Due to flocculation (particle aggregation), suspended particles and colloids are expected to settle out of suspension close to the source, e.g., the river mouth^11^,^14^,^15^. However, significant amounts of labile Fe may extend far offshore^16^ and the particulate fraction is speculated to be important for this transport^3^,^6^,^16^. Fe precipitation kinetics are rapid, with a large proportion of colloidal Fe forming aggregates within minutes of estuarine mixing^15^,^17^,^18^,^19^. The availability of organic matter may considerably affect the solubility of Fe, thus Fe precipitation, in the meltwater system^17^,^20^,^21^. Nutrients supplied by meltwater are drivers of marine carbon cycling due to the primary productivity in Arctic coastal waters and may account for up to 97% of the total CO_2_-drawdown in estuarine regions in the Arctic^22^. In the Antarctic region and entire Southern Ocean it is well established that Fe is a key limiting nutrient for the primary productivity^23^, but the major supplies of Fe are still debated^24^. The meltwater flux to the coastal seas of the southern part of Greenland has increased by almost 50% during the period from 1992–2010^25^,^26^, which has increased the flux of micronutrients, e.g., Fe. This is expected to increase in the future^3^. The suspended matter and nutrients supplied by meltwater are transported from the mouth of meltwater rivers in high-concentration, low-salinity plumes (meltwater plumes). Efforts to quantify these plumes using remote sensing techniques have shown a general coupling between meltwater supply and yields of suspended particulate matter^27^,^28^. However, the relationship is non-linear and is river system-specific^28^ as remote sensing data only observes a snapshot of the water surface and not the vertical extent, currents, advective transport below the surface or particle processes in and below the plumes.

To this date, it is unclear whether significant amounts of Fe may be transported far off-shore in the Arctic context^29^. Furthermore, flocculation processes are not yet well understood in the polar regions, although it is well appreciated that suspended particles in river plumes flocculate and Fe precipitation coupled with minerogenic sediment flocculation is argued to be important^16^,^30^. Arctic rivers are intermittent and characterized by discharges that are highly event-driven and diurnally fluctuating with often very short transport paths from the glacial source to the estuary. These characteristics are all in contrast to most other rivers of the world; hence the processes and fluxes are not the same and Arctic meltwater plumes will have a large potential for transport of e.g. labile Fe. An understanding of particle processes is necessary to predict the availability of Fe and other nutrients and colloids in the polar regions because the kinetics of the flocculation and co-precipitation of Fe, for example, have an impact on the densities and settling velocities of the formed flocs.

Here, we focus on the importance of the labile Fe supplied to estuarine waters in a meltwater plume and its potential transport to the sea by examining a fjord system with estuarine circulation. This study examines the influence of the availability of labile Fe on particle transport processes and the Fe cycle in an Arctic context based on a combination of field observations in Disko Fjord, West Greenland, and laboratory experiments. In the Disko Bay area, nitrogen has been shown to be the limiting factor for the spring bloom^31^, and nitrogen is known to be closely linked to carbon; through these relationships, nitrogen is also linked to other elements, including Fe and aluminium^1^. Thus, the availability and reactivity of Fe may influence the entire cycle of nutrients and solutes, particularly through flocculation, and this must be taken into account when assessing possible distribution patterns and budgets of the solutes and nutrients. The majority of the bedrock of Disko Island is Paleogene basalt in contrast to the Precambrian gneiss dominating much of the mainland^32^, and the island may therefore serve as an example of a terrestrial hotspot for Fe supply to the area. Furthermore, due to the confined morphology of fjords and the multitude of fjord systems in the Arctic, Disko Fjord is an ideal site to study the dynamics of particles and exported matter redistribution in Arctic meltwater plumes. The fjord was visited during two summers and *in situ* measurements were made of particle sizes (in equivalent spherical diameters, ESD) and settling velocities from settling tubes in combination with water samples to determine the mass of the suspended particulate matter (SPMC) and the concentration of particulate labile Fe in the form of reactive and amorphous Fe oxides (Fe_RP_). The Fe_RP_ is extracted from the particulate matter remaining on cellulose filters (0.45 μm retention diameter) after water sample filtration using an oxalate solution, which has been shown to extract amorphous Fe oxides from glacial meltwater sediment well^33^ (see Methods). Systematic laboratory experiments have been carried out in which the concentration of available dissolved Fe was increased to test the flocculation kinetics and floc shape dynamics based on various treatments. For all measurements, a laser sheet camera system has been used to determine particle sizes and shapes (see Methods).

## Results

**Figure 1:**
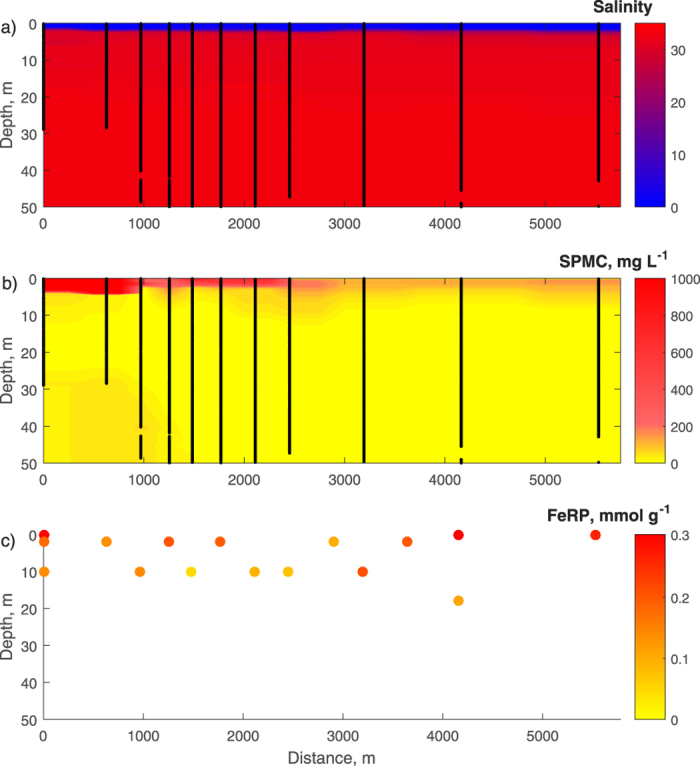
Transect on the 8th of August 2014. Distance is from the river mouth into the fjord. Black lines in (**a,b**) show locations of instrument sampling upon which the interpolated values are based. SPMC is calibrated turbidity measured together with the salinity. The Fe_RP_ values in (**c**) were measured from 1 L water samples (see methods section) collected at the locations of the dots. The water depths at the two innermost stations are 30 metres; the water depths at the other stations are >50 metres. Supplementary Fig. 4 shows the same three panels as Fig. 1 as well as a panel showing the [Fe_RP_] normalized to the filtered volume of water.

A 6 kilometre transect measured in August 2014 provides an example of the extent of the plume in Disko Fjord (Fig. 1). The fjord is highly stratified, with very little vertical mixing of water bodies. The low-saline plume is approximately 2 meters thick in the entire transect and the sediment plume is also located in the top few metres of the entire transect. No change in the salinity is seen in the plume, and while the SPMC is highest in the inner part (≥1000 mg L^−1^), the entire plume transect is associated with elevated SPMC (≈200 mg L^−1^). [Fe_RP_] is consistently higher in the plume (0.26 (±0.06) mmol g^−1^) than below the plume (0.1 (±0.03) mmol g^−1^), and the concentration stays in the same range throughout the plume. The concentrations are in the range of the 0.02–1.0 mmol g^−1^ of dithionite extractable Fe oxides determined from various meltwater regions^1^. The total dissolved Fe concentration, [Fe_TD_], was consistently below the laboratory assessed detection limit (0.1 μM), thus in the range of previous studies that have reported values ranging from 0.02–2.9 μM in other Greenlandic glacial meltwaters^2^,^3^,^6^. The suspended matter is characterised by very low settling velocities with six of seven measurements below the analytical lower limit of 0.012 mm s^−1^, equivalent to one metre of settling per day (Supplementary Table 1) and corresponding to a Stokes’ settling velocity of a quartz grain with an ESD of 4 μm. The transect was measured during ebb tide with very little wind, and horizontal current speeds were on the order of 0.1 m s^−1^. The settling velocity is controlled by the size, density and shape of flocs, which range in mean ESD from 50 to several hundred microns, compared to the mean primary particle ESD of the individual particles, which is approximately 10 microns. The low settling velocities observed here are explained by the complexity in the shapes of the flocs. As an example, the mean sphericity of flocs in the upper 2 metres of the entire plume is significantly lower than the mean sphericity of particles at 5 metres depth close to the river mouth (Supplementary Fig. 3).

**Figure 2:**
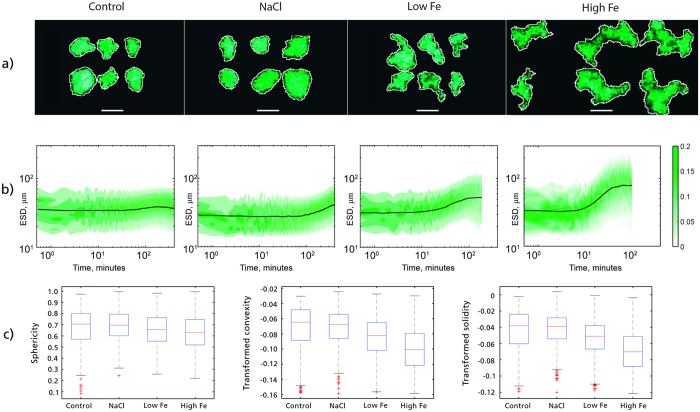
Treatment-specific differences in particle properties. The four columns in (**a,b**) each represent one specific treatment type (control, NaCl, Low Fe and High Fe). Six example treatment-characteristic particles (**a**) are shown together with the change in the frequencies of the entire particle size distribution over time (**b**) and box plots of three shape parameters based on measurements of the suspended particles in each treatment (**c**). Supplementary Table 3 shows the change in temperature, pH and conductivity from start to end during the experiments. The scale bar in (**a**) is 100 μm long and the same for all four columns. The color bar in (**b**) shows the frequency of occurrence (fraction of total number) of a given particle size. Convexity and solidity in (**c**) has been transformed to normality using BoxCox-transformation^53^. The red line is the median, the box shows the inter-quartile range and the whiskers indicate the largest and smallest values with the exclusion of outliers (red crosses), which are values outside +/−2.7 standard deviations. The ANOVA tests in Supplementary Fig. 6 show that the Control/NaCl, Low Fe and High Fe treatment shapes are significantly different (p < 0.001), whereas the Control and NaCl shapes are not significantly different.

The systematic effect of Fe on particle properties has been demonstrated in a laboratory study (Fig. 2). Flocs were formed in a suspension tank by adding sediment from the fjord an applying four different treatments (see Methods for elaborated description). One treatment was a control treatment where the sediment was added in fresh water, one treatment was a test of the effect of salt ions by adding NaCl, and in two treatments dissolved Fe was added at concentrations of 4 (Low Fe) and 26 (high Fe) μM. The low Fe concentration is in the range of what is measured in meltwater rivers today^3^,^6^ and the High Fe treatment is made to exemplify how flocs form and take shape when they incorporate relative large amounts of dissolved Fe. Flocs from the control and NaCl treatments have relatively high sphericities, on the order of 0.8, and still seem somewhat elongated, whereas flocs formed when dissolved Fe is present are more irregular, with lower sphericity, convexity and solidity (Fig. 2a,b). ANOVA tests (Supplementary Fig. 6) show that the floc shape parameters sphericity, solidity and convexity decrease significantly with increasing Fe availability. Lower sphericities and solidities are indications of lower densities, whereas a lower convexity increases the specific surface area of the flocs.

The concentration of added dissolved Fe has a marked influence on the kinetics of the flocculation process as well as the resulting floc properties. The initial mean ESD was 14.0 μm (+/−3.7; Supplementary Fig. 7), and all treatments show an increase in the mean ESD over time. After sediment is mixed in the NaCl solution, flocculation is initiated, as demonstrated by a slow but steady increase in mean ESD over time. Adding a small amount of dissolved Fe instead (the low Fe treatment) slightly increases the maximum ESD reached relative to the NaCl treatment. More importantly, the time needed to reach the maximum floc size is markedly shorter, decreasing from approximately 6 hours to approximately 90 minutes. Adding more dissolved Fe (the high Fe treatment) results in increases in the maximum ESD reached, and the time taken to reach the maximum diameter decreases. [Fe_TD_] decreases, while [Fe_RP_] correspondingly increases (Supplementary Fig. 5). This fits with the results of Boyle*, et al.*^14^, who observed precipitation of dissolved Fe within hours, and Nowostawska*, et al.*^17^, who showed that most colloidal Fe is aggregated within seconds.

**Figure 3:**
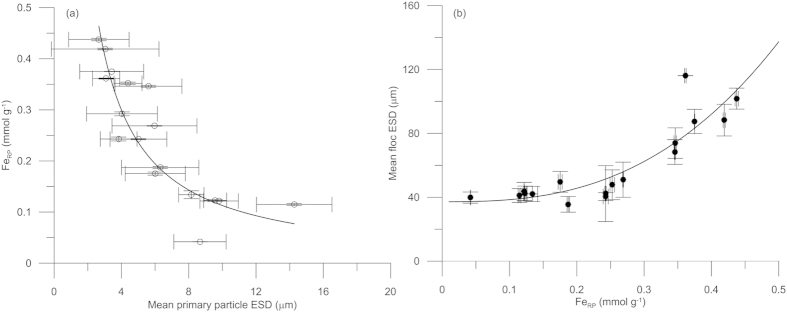
Relationships between Fe concentration and particle/floc diameters. Water samples from the fjord in 2013 show that [Fe_RP_] is a function of primary particle ESD (**a**) and that floc ESD is a function of [Fe_RP_] (**b**). Error bars indicate one standard deviation of the mean. [Fe_RP_] decreases with increasing primary particle ESD, and floc ESD increases with increasing [Fe_RP_]. The two power regressions are highly significant (p < 0.01).

Dispersed particles from *in situ* water samples show that the [Fe_RP_] in the primary particles is dependent on the diameter of these particles (Fig. 3a). The relationship is highly significant (p < 0.01) and shows a decrease in [Fe_RP_] with increasing primary particle diameter, which is caused by the larger specific surface area of the smaller primary particles^1^,^33^. The mean *in situ* floc ESD of the same samples shows a highly significant (p < 0.01) relationship, in which the floc diameters increase with increasing [Fe_RP_] (Fig. 3b).

## Discussion

The laboratory study shows that the kinetics of the flocculation process clearly is linked to the availability of labile Fe and increased levels of Fe increase the surface area of the flocs while simultaneously causing lower densities. The *in situ* samples show that larger flocs are composed of small primary particles and incorporate more Fe_RP_. This is in agreement with the flocculation experiments showing that the flocculation kinetics and diameters reached are closely linked to the availability of labile Fe. The particles that stay in suspension in the plume are less spherical, indicating the influence of the shape and character of the flocs on the horizontal transport potential. Very low settling velocities equivalent to that of a quartz sphere of only 4 μm diameter prevent the particles from settling out and keep them in suspension in the plume for a prolonged period of time and in the presented example particulate matter is transported several kilometres outwards without settling more than a metre.

The theoretically expected increase in settling velocities with increasing ESD is not observed (Table S4). This deviation from what is expected can now for the first time in an Arctic context be assessed in relation to the shape and character of the flocs under both field and laboratory conditions. The results show a strong correlation between [Fe_RP_], floc size, floc form and properties controlling the fate of the meltwater-supplied sediments and associated substances. Several studies in the past have focused on how the particulate fraction of Fe is significantly greater than the dissolved fraction and how this particulate fraction may be transported far from the source^2^,^5^,^6^,^7^,^16^. However, these studies did not include the direct links between the suspended particulate matter, meltwater plume extent and flocculation dynamics. The *in situ* observations from Disko Fjord in combination with laboratory sensitivity studies demonstrate the importance of Fe on longer travel distances. We identify a positive feedback in that the horizontal transport of Fe and sediment is dependent on the associated flux of Fe from meltwater rivers because the flux of Fe from the meltwater river system to the fjord environment drives the flocculation. Thus, even though a major part of the supplied matter might be deposited close to the source due to changes in flow patterns, a substantial amount of flocs with high Fe content is transported horizontally away from the source area, which is possibly enhanced by highly stratified estuarine conditions. This may have great implications for the cycle of Fe and other related substances and together with the large Fe availability from the basalts of Disko Island may be a key reason for why the primary productivity, for example in the Disko Bay area, has not been shown to be Fe limited^26^. It underlines the importance of hotspots and meltwaters on larger regions in the Arctic. Furthermore, it shows that the direct interaction between labile Fe and particles is important to keep in mind when assessing the Fe fluxes today and in the future both in the Arctic and in the Fe limited Antarctic region.

In a future climate perspective, we expect the amount of Fe and other solutes reaching the aquatic environment to increase due to greater meltwater discharge and event-driven pulse fluxes^10^,^34^, which may increase erosion and transport rates. Although dilution might also occur, our assumption is that more Fe will thus be released to fjords through meltwater rivers. Our *in situ* and laboratory studies show that the particle-bound labile Fe promotes flocculation, but the decreased sphericities and solidities, which result in lower settling velocities for the resulting flocs, restrict deposition of the suspended material. Therefore, there are clear indications that increased supply of Fe will not result in more rapid deposition of suspended material. Instead, a similar increase in the export of labile Fe and related substances must be expected. Due to the increased horizontal flux of suspended particulate matter from meltwater systems in the future, the fluxes of matter to the marine environment will thus increase. Disko Fjord is just one of numerous outlets from Disko Island^35^ and just one example of an Arctic fjord system. Thus, areas like the Disko Island environment are and may continue to be a regional hotspot for the supply of nutrients and solutes from the terrestrial to the coastal marine environment. The results presented here must also be valid for other Fe-rich Arctic coastal environments that will experience increasing fluxes of matter. Furthermore, the clear link between flocculating suspended particles and labile Fe is important to address in the context of the Fe-limited Antarctic region and Southern Ocean. We demonstrate how flocculation involving labile Fe alters the fluxes of meltwater transported particles and iron, which thereby may increase the fluxes away from the source area. By that we challenge the classical assumption of estuarine removal^14^ of labile Fe by showing that flocculation does not only increase sedimentation rates in estuarine environments. Our Fe results are based on concentrations of labile, oxalate-extractable amorphous Fe oxides. Clearly, the reactivity of Fe oxides is important, and the reactivity is highly dependent on the mineralogy which again is dependent on the source as well as the weathering processes involved. Future studies should be conducted to try and assess the quantitative implications of the alterations we find, and furthermore examine the alterations in relation to the different meltwater transport processes and sources in polar regions. Finally, Fe precipitates are known to be carriers of other elements and nutrients; thus, future investigations appear to be needed to further increase our understanding of the role of flocs and flocculation in nutrient and solute cycling and its control of marine primary productivity in polar regions.

## Methods

### Field site

Disko Island (West Greenland) is in the transitional zone between the low and high Arctic. The mean monthly air temperature varies from −16 to 7.1 °C, and the mean annual precipitation at nearby Arctic Station is 436 mm, as measured during the period from 1991–2004^36^. The bedrock of Disko Island is primarily plateau basalts, which are part of a large lava basin extending along the entire western margin of continental Greenland^37^. Most of this basin is situated offshore and is only exposed to weathering in the area around Disko Bay^38^, making the island a hotspot for labile Fe from the eroding basalt. The Sermerssuaq Ice Cap is the major ice cap on Disko Island, and an outlet glacier connects the ice cap to the north-eastern part of the Disko Fjord (Kangerdluk) called Kuannersuit Sulluat, where sampling took place (see Supplementary Fig. 2). The outlet glacier was surging from 1995–1999, delivering large amounts of meltwater and sediment to Disko Fjord^39^. Today, it is retreating and the proglacial valley has extended, providing a supply of sediment and material both from sub- and supraglacial sources as well as from river bank and topsoil erosion in the proglacial valley. The meltwater river is braided in the lower part close to the fjord head, making river discharge measurements difficult, but discharges measured in the late 1990s were on the order of 35–100 m^3^ s^−1^ with sediment loads of several g L^−1 ^39. The catchment area of the Kuannersuit Sulluat fjord is 531 km^2 ^40. The depth of the fjord increases quickly to 30 m depth near the delta front and gradually to a maximum depth of 120 m and a width of approximately 2 km over the next 20 km. The fjord is tidally influenced by semi-diurnal tides and features a spring tide range in the inner part of the fjord of 2 m and a maximum range of 2.5 m^41^. The fjord is ice-covered during the winter but was completely ice-free during the two summer sampling seasons in 2013 and 2014.

### Water samples

Profile measurements were carried out using a YSI 6600 V2-4 CTD probe with a turbidity sensor and two particle sizers, a LISST and a Pcam (see below). The profiling speed was 1 m/s. Water samples were taken *in situ* using a 5 L Niskin water sampler in which the water was thoroughly stirred. Two 1 L subsamples were transferred into separate pre-rinsed polyethylene bottles. The samples were immediately filtered on board the vessel through one 47 mm Whatman GF/F glass fibre filter with a 0.7 μm retention diameter and one 47 mm Advantec nitrocellulose filter with a 0.45 μm retention diameter. The first 100 mL of the nitrocellulose filtrate was filtered directly into a new 100 mL polyethylene bottle and frozen for later analysis in the laboratory. The two filters were also frozen for later analysis and laboratory blanks were made. In the laboratory experiments, 200 mL water was extracted for each filter, and the filtration process was the same as the one applied onboard the vessel except that Millipore cellulose filters were used instead of Advantec filters. Sampling with settling tubes was carried out in order to determine the median settling velocity of the suspended matter. Primary particle size measurements were carried out in the laboratory by ultrasonically dispersing (2 minutes on a Bandelin UW 2200) sediment from the cellulose filters in a sodium pyrophosphate-solution and analysing the dispersed particles on a Malvern Mastersizer E/2000.

### Fe extraction

The concentrations of total dissolved iron, Fe_TD_, and the particulate iron fraction considered to be highly reactive and labile, Fe_RP_, were measured. Fe_TD_ is defined as the fraction that passes through a 0.45 μm retention filter and is measured in triplicate by atomic adsorption spectrophotometry, AAS (Perkin Elmer AAnalyst 400), which in our lab tests has shown to provide usable concentrations in the order of 0.1 μM. Fe_RP_ was determined from frozen cellulose filters after they had been dried for 4 hours at 60 degrees C, left to adjust to room temperature for one hour and weighed to determine the particulate mass. We note that the freezing and heating may have slightly reduced the solubility of some amorphous oxides^42^. The Fe_RP_ was extracted from the filters following the method described by Mckeague and Day^43^ and successfully applied on glacial meltwater sediments by Poulton and Canfield^33^. An oxalate solution was prepared using 0.114 M ammonium oxalate mixed with 0.086 M oxalic acid to achieve a 0.2 M solution with a pH of 3, and the amorphous Fe oxides are extracted after filters have been shaken for four hours in 10 mL of the oxalate solution. The extract is filtered into a 20 mL polyethylene vial and the dissolved Fe is measured by AAS after calibration with Fe standard solutions. Laboratory blanks were always made to correct the results of the AAS. The relative content of amorphous and crystalline Fe oxides was determined by subjecting samples in pseudo-triplicate to first an oxalate extraction procedure as described above followed by an extraction using dithionite as described in Poulton and Canfield^33^. Of the total extractable oxides (the sum of oxalate and dithionite), 20% (±1.6) was extracted with dithionite after extraction with oxalate, showing that the labile oxalate extractable oxide pool consists of 80% (±1.6) of the total Fe oxide pool.

### Suspended particle characterisation, LISST

Two instruments were used to estimate the size of particles in suspension. Both instruments can be used *in situ* or in a laboratory setup and their main function is to estimate the particle size distribution (PSD) of particles suspended in a water column. The LISST-100C (Laser *In Situ* Scattering Transmissometry) is a laser scattering instrument that has been used in many *in situ* estuarine studies^44^,^45^,^46^,^47^,^48^. The instrument emits a laser beam over a 5 cm path and measures the forward scattering signal by suspended particles in 32 logarithmically spaced ring detectors^49^. The angle is inversely proportional to the size; thus, larger particles are associated with smaller dominant scattering angles. Through inversions using an updated kernel based on Mie theory, the scattering signal is converted to a PSD^50^. This kernel is based on randomly shaped surfaces on overall spherical particles, meaning that the LISST assumes the particles to be spherical. The size is represented by the equivalent spherical diameter (ESD) and the range is from 2–400 μm.

### Suspended particle characterisation, Pcam camera system

A new camera system, Pcam, was developed at MARUM, Bremen University, as an updated version of the DISDAL^51^. The Pcam is a submersible laser sheet camera system. Starting in 2014, the system incorporates a Canon D70 digital SLR camera with a 60 mm Canon EF-S macro lens inside a pressure housing. The pixel size of the camera is 4.1 μm and pictures are taken in 1:1 aspect ratio, giving the camera in this setup a lower size limit for particle detection of approximately 20 μm and an upper size limit of several mm. The 2013 version of the instrument had a Canon D60 camera with a pixel size of 4.5 μm. Pictures are illuminated by a green, collimated laser sheet thereby avoiding three dimensional distortion effects and decreasing the influence of out-of-focus particles. See supplementary information for a description of image processing.

The correlations between the changes in mean ESD over time of the LISST and of the Pcam are highly significant (p < 0.0001; Supplementary Fig. 7 and Supplementary Fig. 8 and Supplementary Table 4), demonstrating that the two methods provide similar results with regard to particle sizes, despite minor deviations during the initial phase of the treatments due to differences in the particle size range between the two instruments.

### Laboratory experiments

Flocculation experiments in a controlled laboratory environment were carried out in spring 2014. The experimental design consisted of a 50 L container (W × H × L of 55 × 30 × 30 cm) with a small 10 cm rotating propeller. The propeller was controlled by a variable power supply and was set to stir at 30 rpm in the bottom of the container to simulate a low turbulence that keeps slower settling particles in suspension. The starting temperature of the water was 10 degrees C, which has been observed to be a common temperature of the surface water in the Disko fjord on a sunny summer day. Four different treatments were applied, three in which fresh (tap) water was not altered and one in which NaCl was added to achieve a salinity of 5% (see Supplementary Table 2). Tap water from the same ground water source was used for all experiments with no initial alterations other than oxygenation. The pH of the source was 7.5, the conductivity was on the order of 1 μS cm^−1^, and the natural concentration of dissolved iron ranged from 0.18–0.54 μM. The salinity in the NaCl treatment was 5 PSU because it has been shown that salinities above a few units do not increase the influence of the salt ions on flocculation^52^. The pH and conductivity was monitored and recorded throughout the experiments. After filling the tank with the fresh or NaCl-supplemented water, 2.5 grams of ultrasonically dispersed (2 minutes on a Bandelin UW 2200) sediment was added and thoroughly mixed throughout the entire water column, yielding a starting SPMC of 50 mg L^−1^. Rotation of the propeller in the bottom of the tank was started. In two of the three fresh water treatments, dissolved iron was added from an Fe(NO_3_)_3_ nitric acid solution with an Fe concentration of 0.018 M (1000 mg L^−1^) immediately before adding the sediment. The concentration of added dissolved Fe was either 4 or 26 μM, the first being an example of the high range of concentrations found in the field^3^,^6^ and the latter being an example of a very high concentration, representative of events or hotspot areas. As soon as each experiment was set up and everything was well mixed, the Pcam and/or LISST instruments were started, measuring at intervals of 30 seconds. The sediment used in the treatments was collected from the bottom of the inner part of Disko Fjord, West Greenland, in July 2013 by a gravity corer. Sediment from the top 10 cm of the core was removed, mixed and freeze-dried prior to use. The sediment is fine grained and has a mean ESD of 10 μm and <6% coarse grained material, based on measurements on the Malvern Mastersizer E/2000.

## Additional Information

**How to cite this article**: Markussen, T. N. *et al.* Flocculated meltwater particles control Arctic land-sea fluxes of labile iron. *Sci. Rep.* **6**, 24033; doi: 10.1038/srep24033 (2016).

## Supplementary Material

Supplementary Information
