# Supplementary material for: Flocculated meltwater particles control Arctic land-sea fluxes of labile iron
Source: Sci Rep. 2016 Apr 6;6:24033. doi: 10.1038/srep24033 (PMC4822144; doi:10.1038/srep24033)
Supplement: Supplementary Information [file srep24033-s1.pdf]

Supplementary:

Flocculated meltwater particles control Arctic land-sea fluxes of labile iron

Thor Nygaard Markussen[1], Bo Elberling[1], Christian Winter[2] & Thorbjørn Joest Andersen[1]*

[1]Center for Permafrost (CENPERM), Department of Geosciences and Natural Resource Management, University of Copenhagen, Øster Voldgade 10, DK-1350 Copenhagen, Denmark

[2]Center for Marine Environmental Sciences (MARUM), University of Bremen, Leobener Str., D-28359 Bremen, Germany

Table of Contents

Supplementary methods 3

Image processing 3

Supplementary figures and tables 6

In situ measurements 7

Flocculation experiments 9

Pcam performance compared to that of LISST 11

Supplementary methods

Image processing

The images are processed in Matlab using a script with four steps and involving several functions from the Image Processing Toolbox. First, a morphological reconstruction technique is applied to filter out noise and out-of-focus particles. In morphological reconstruction a marker image is created based on the input image, called the mask. In the marker image, all intensities of the mask image are subtracted by a constant number. The marker image is then dilated, meaning that the intensities are gradually increased, but the intensities of individual pixels can never be higher than they were in the original mask. This will identify peaks in the image, which in this case are in-focus particles. In this way, low-intensity objects too far from the camera, i.e., out-of-focus particles, are removed without changing the intensity or shape of particles that are a combination of low- and high-intensity features. The second step is to convert the image to a binary image. This is based on a relative intensity threshold that is 0.1 of the maximum intensity of the morphologically reconstructed image. Thus, pixels with intensities above 0.1 times the maximum intensity will be considered to be pixels containing information about particles. It is necessary to use a threshold as lightning distortion effects cannot be eliminated and will create erroneous particles and shapes if they are not removed. In most cases, the maximum intensity of an image will be 255, but in some cases it might be lower due to differences in the water properties and small electrical irregularities. Numerous tests have shown that the applied threshold yields good results across environments and deployment configurations. Individual particles are found based on the binary image using the "regionprops" function in Matlab, but particles with a maximum intensity that is less than half the overall maximum intensity are removed because these particles are not in focus enough to yield reliable information. An example of an input image is shown in Fig. S1a, and the identified particles are shown in Fig. S1c. Shape parameters, area and the equivalent spherical diameter (ESD) are computed based on the remaining particles. The ESD of one particle is the corresponding diameter of a circle with the area of the illuminated area of the particle. A PSD

for each image is calculated and the mean ESD is calculated based on the method of moments. Fig. S1b shows the calculated PSD and mean ESD based on the example image.

Particle shape and porosity characteristics aid in understanding the properties of individual flocs and can be an indicator of relative differences in the specific surface area of flocs. Shapes can be estimated based on the images, assuming that the shape in the 2D image represents the three-dimensional shape. The three characteristics of sphericity, convexity and solidity are used here. Sphericity is the measure of the ratio between the major and minor axes of the best-fitting ellipsoid and is thus 1 for completely spherical particles and decreases towards 0 for increasingly rod-like or elongated particles. Convexity is a parameter often used in grain characterisations to describe the roughness of the surface of the particle. It is measured as the ratio, with a value of 0 to 1, between the perimeter of the convex hull, i.e., the smallest polygon inside which the object fits, of the particle and the perimeter of the actual particle. A convexity of 1 means that the surface of the particle is completely smooth, and convexities closer to 0 indicate longer particle perimeters in relation to the convex hull perimeter, indicating a rougher surface. Note that convexity is normally measured using a high-resolution microscope image, and the 4-µm resolution of the Pcam cannot be used for the small particles. However, for larger particles above ~100 µm, the convexity measured with the Pcam can provide an estimate of the relative difference between particles. Thus, it is not a direct measure of the surface roughness. The solidity provides an estimate of the overall fluffiness or porosity of a particle and is the ratio between the particulate area and the convex hull area. The boxplots in Fig. S1d depict the acquired particle shape information based on the example image.

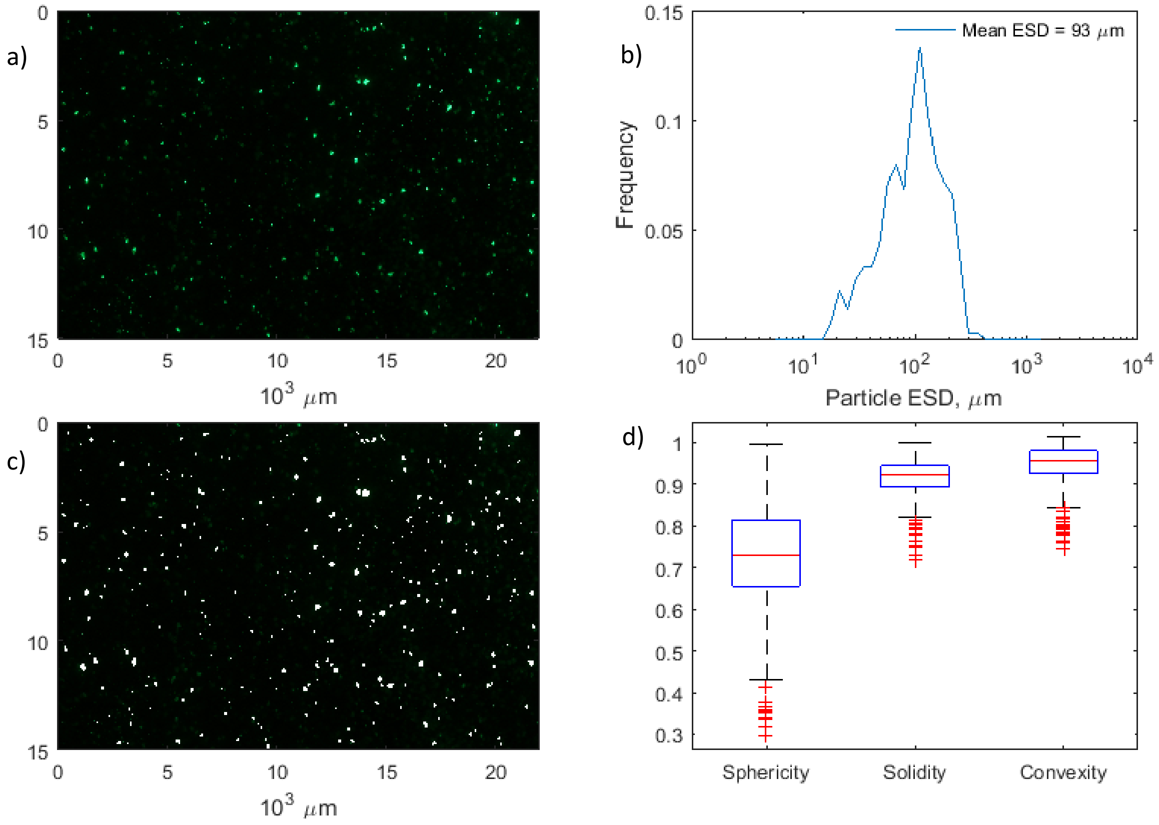

Figure S1. Example of the Pcam data. Image of flocs captured by the Pcam (a) and those selected after processing (c). The processing results are shown in the form of a PSD and mean ESD (b) and a quantification of particle shapes presented in a box plot (d). In the box plot, the red line is the median, the box shows the inter-quartile range, and the black whiskers show largest and smallest values, excluding outliers (red crosses), which are values outside +/- 2.7 standard deviations.

Supplementary figures and tables

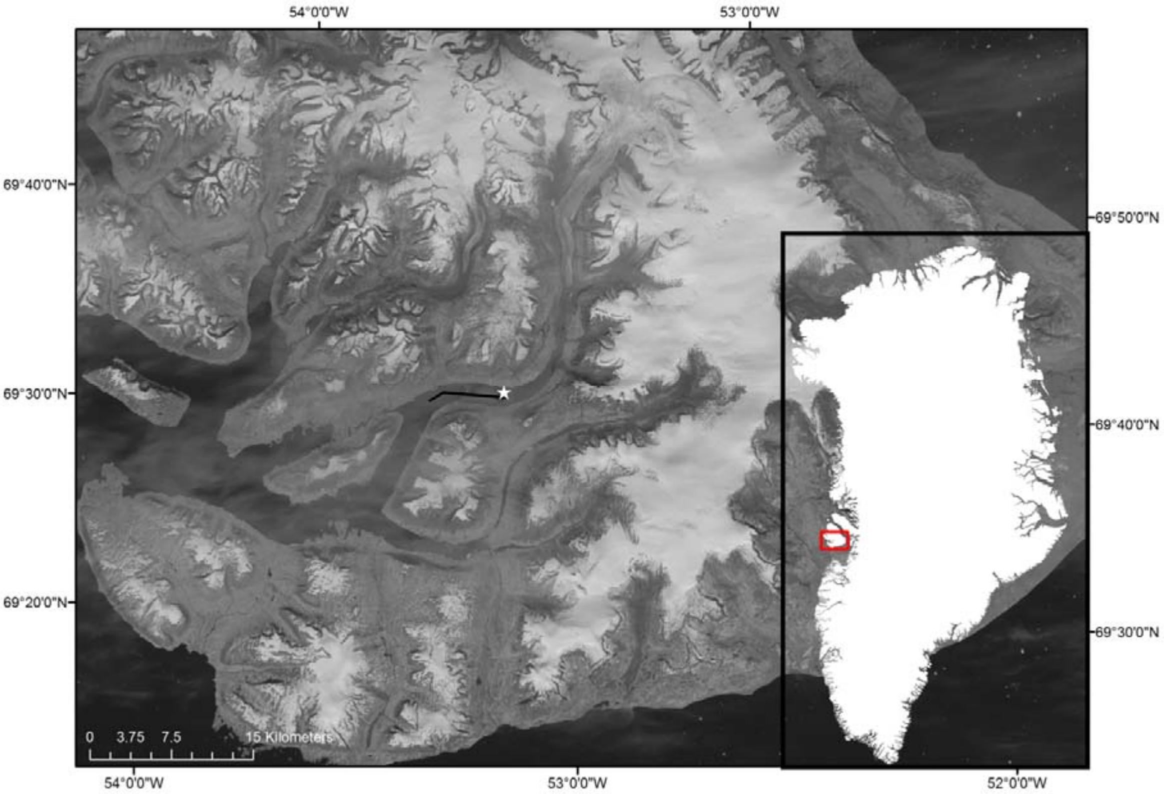

Figure S2. Overview of the study area and field site. The inset shows the location of Disko Island (red box) in West Greenland. The black line on the main map shows the location of the transect (see Fig. 1, main text), the white star marks the location of sampling close to the river outlet. The glacier terminus is roughly 10 kilometres from the river outlet. The background image is a grey/white Landsat 5 grayscale satellite image from July 2001 and the map in the inset is a simple coast outline. The figure was created using ArcGIS, version 10.1, http://www.esri.com/software/arcgis/.

In situ measurements

ID	Distance from river mouth (km)	Depth of measurement (m)	SPMC (mg L^{-1})	ESD (µm)	Ws (mm s^{-1})
**ST1**	0.5	2.5	1100	45	0.1561
**ST2**	1	13	244	-	< 0.012
**ST3**	1	2	606	-	< 0.012
**ST4**	1.2	2.5	292	33	< 0.012
**ST5**	3.5	3	35	31	< 0.012
**ST6**	4	2.5	56	85	< 0.012
**ST7**	10	2	41	72	< 0.012

Table S1. Settling tube results from the 2013 campaign. Seven *in situ* settling tube measurements produced results with settling velocities that are lower than 0.012 mm s^{-1} in all but one example. The SPMC is the combined weight of the ten filters used for the settling tubes, and the ESD, where available, was measured by the Pcam. The lower analytical limit of the settling tube with the used method is 0.012 mm s^{-1}; thus, if settling is measured but a mean settling velocity cannot be assessed, the velocity is <0.012 mm s^{-1}. The Fe_{RP} in the top of the water column in 2013 ranged from 0.3-0.5 mmol g^{-1}, thus being in the same range as the measurements from 2014 (see Figure 1, main text).

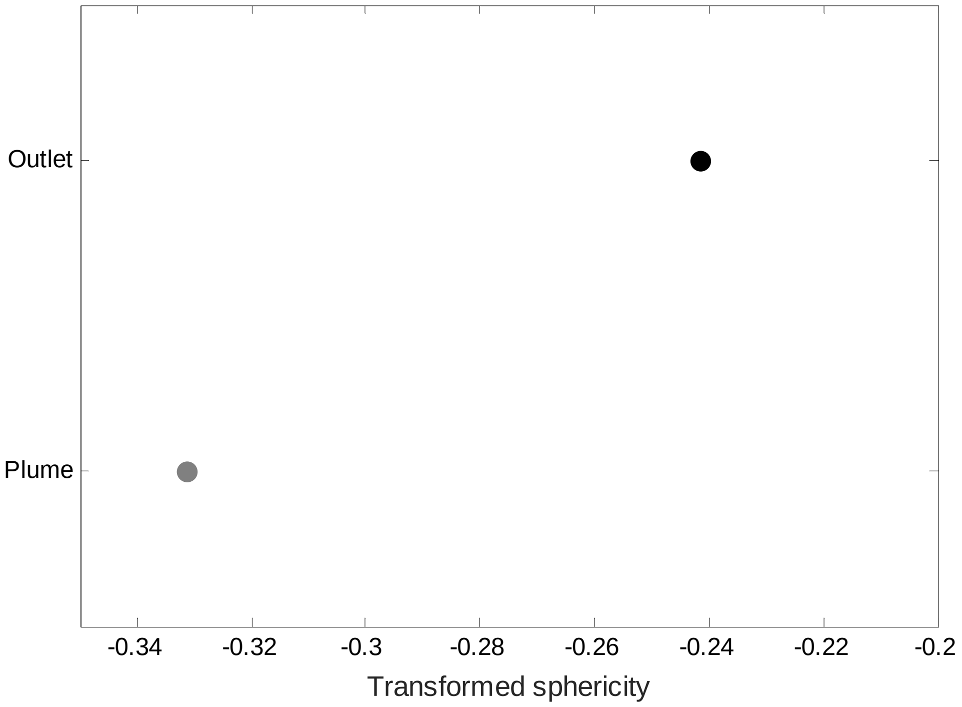

Figure S3. ANOVA test of differences in sphericity (transformed using BoxCox-transformation) between particles in the outlet of the river and those from the plume (measurement depth: <2 m). The analyses are based on the Tukey-Kramer test. The 95 % confidence interval cannot be shown as it is too small. Therefore, plume sphericities are highly significantly lower (p<0.001).

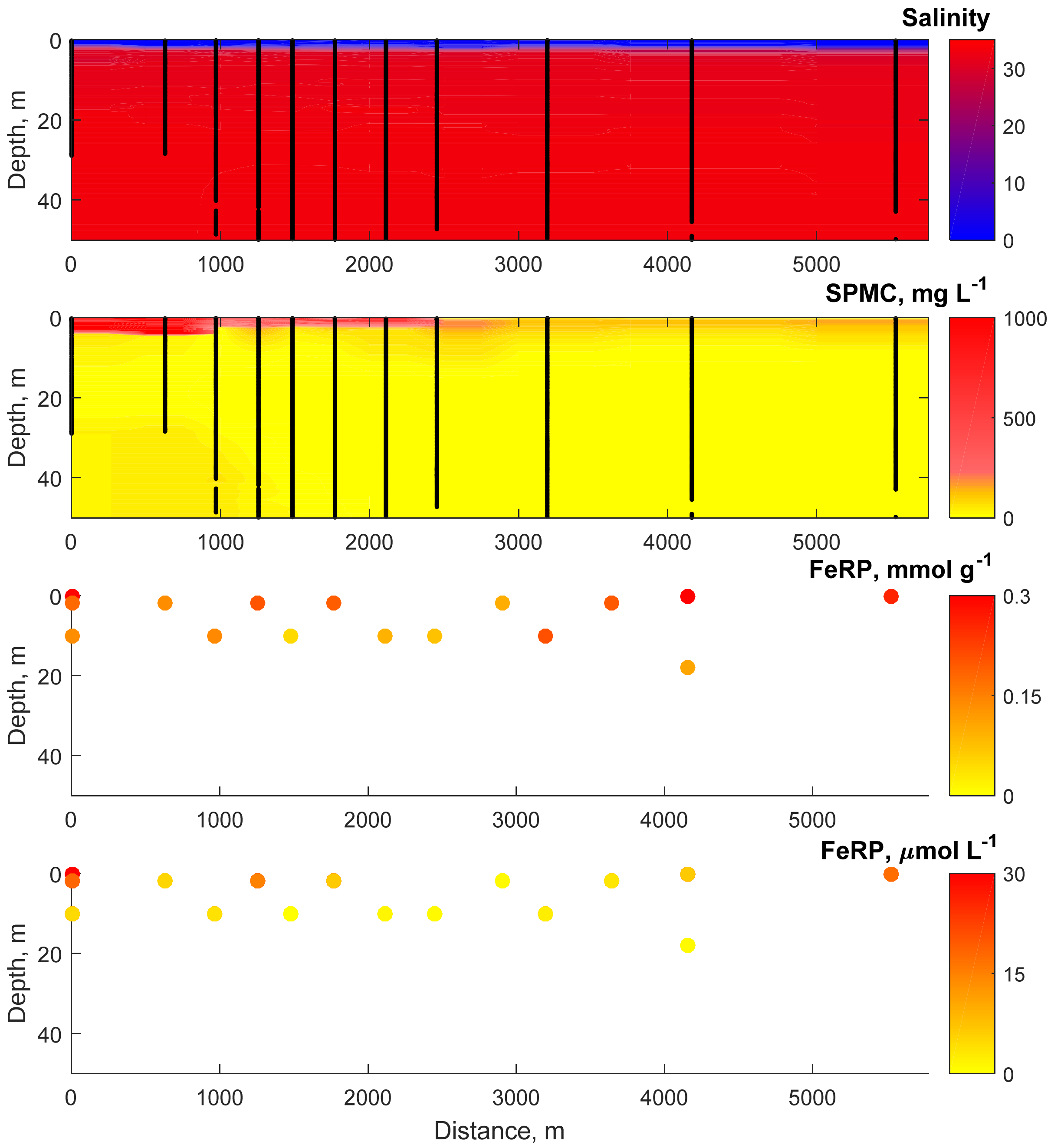

Figure S4. The first three panels of this figure are equal to those in Fig. 1 of the main text. The fourth panel shows the [Fe_{RP}] normalized to volume of water filtered. In the extraction procedure, the Fe is concentrated meaning that the detection limit of Fe_{RP} is 0.01 µM. The range of all measurements of [Fe_{RP}] in the plume during the two campaigns was 1.4-574 µM or 0.1-0.39 mmol g^{-1}.

Flocculation experiments

Treatment	Fe solution added	Fe_{TD} concentration	Salinity
**Low Fe**	10 mL	4 µM	0
**High Fe**	65 mL	26 µM	0
**NaCl**	0 mL	0 µM	5
**Control**	0 mL	0 µM	0

Table S2. The four different treatment types used in the experiments. Fe from the dissolved Fe solution was added in the two different Fe treatments, yielding the initial total dissolved Fe (Fe_{TD}) concentrations shown above.

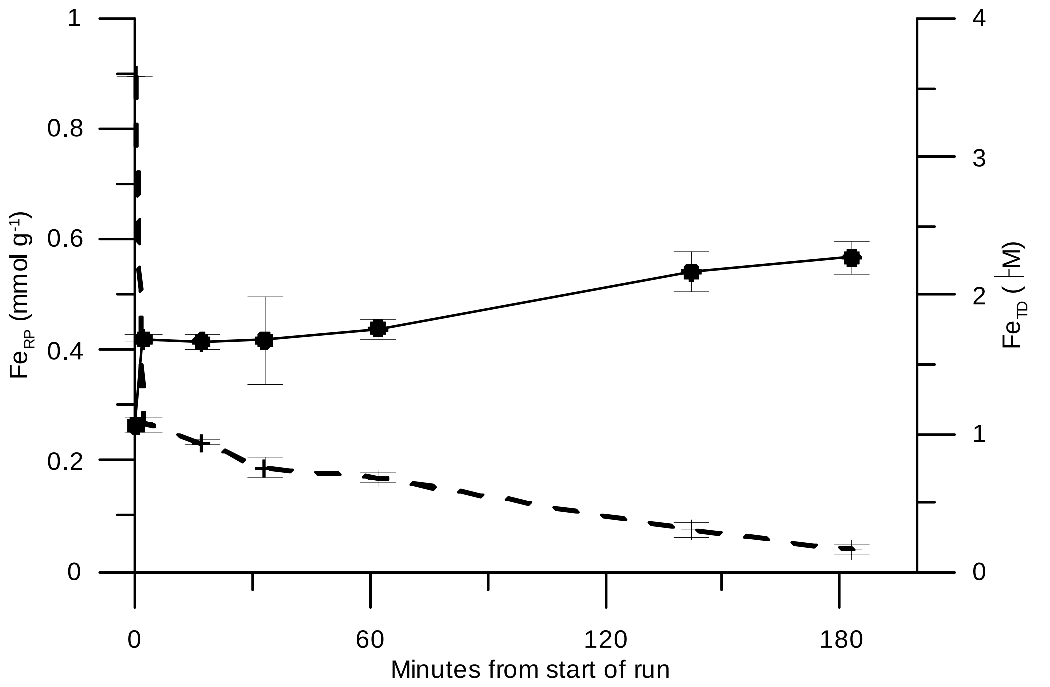

Figure S5. Changes in concentrations of Fe over time. Examples of changes in [Fe_{RP}] (dots and solid line) and [Fe_{TD}] (crosses and dashed line) over time in the low Fe treatment. The [Fe_{TD}] at time zero is the amount of dissolved Fe added at the start of the run, and the [Fe_{RP}] at time zero is the [Fe_{RP}] of the sediment.

Treatment	Temp, start	Temp, end	pH, start	pH, end	Cond, start	Cond, end
**Control**	12.5	16.2	7.62	7.80	17.3	19.1
**NaCl**	11.4	14.9	7.51	7.68	200	199.5
**Low Fe**	9.8	12.1	7.42	7.50	18.3	20.6
**High Fe**	9.5	10.9	7.59	7.18	20.5	20.1

Table S3. Water quality data for the laboratory experiments at the start and end of each treatment type.

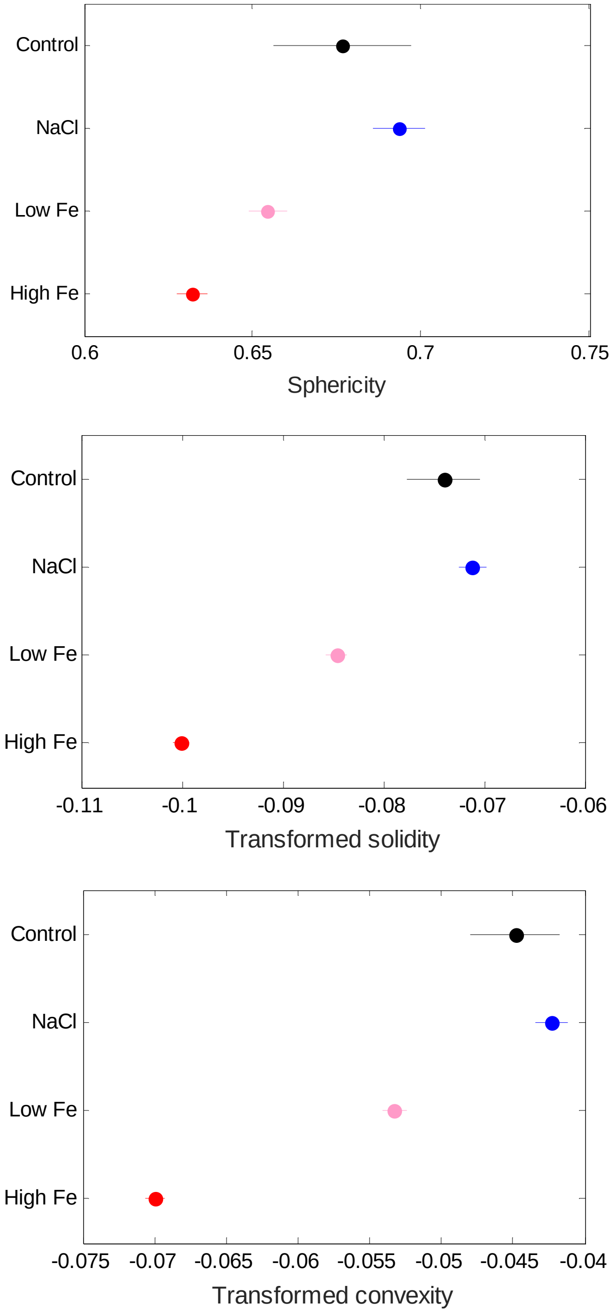

Figure S6. ANOVA tests of significant differences between treatments for the three shape parameters. The analyses test whether the means are significantly different using the Tukey-Kramer test and are based on flocs larger than 100 µm. Circles show the mean value, and lines show the 95 % confidence interval. Solidity and convexity have been transformed using BoxCox-transformation in order for the data to be normally distributed, a requirement for ANOVA analysis. For all three shape parameters, a significant ($p<0.001$) difference is observed between the treatments without added Fe (Control and NaCl), the Low Fe treatment and the High Fe treatment. The differences between the Control and NaCl treatment shapes are not significant.

Pcam performance compared to that of LISST

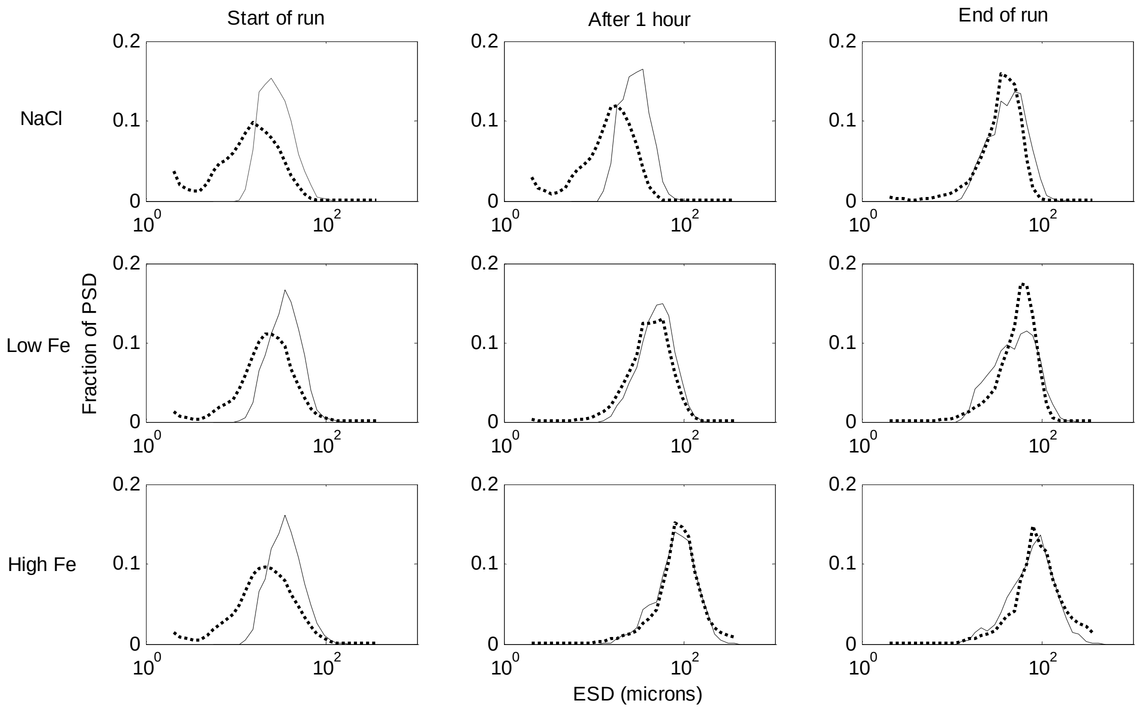

Figure S7. Representative PSDs from the laboratory treatments. Examples from the NaCl treatment (top row), low Fe treatment (middle row) and high Fe treatment (bottom row) at the beginning (first column), after 1 hour (middle column) and at the end (last column) of a run. Solid lines are the Pcam and dashed lines are the LISST. The initial mean ESD of the LISST measurements at the start of the run is 14 µm (± 3.7).

In the early stages of the treatments, the PSD of the LISST has lower values than that of the Pcam. The PSDs from the two instruments are similar after 1 hour in the two Fe treatments, as shown in Figure S, middle column. When the flocculation is at a maximum, at the end of the runs, the PSDs of the LISST tend to show a more well-sorted distribution than the Pcam PSDs (Figure S, right column). The correlation between the mean ESD of the LISST and Pcam for all measurements is highly significant, with an $r=0.95$ ($p<0.0001$). For individual treatments, the statistics show that the correlations are significant, with $r>0.9$ ($p<0.0001$) for all treatments (Table S3). These significant correlations prove that the two instruments show the same trends over time.

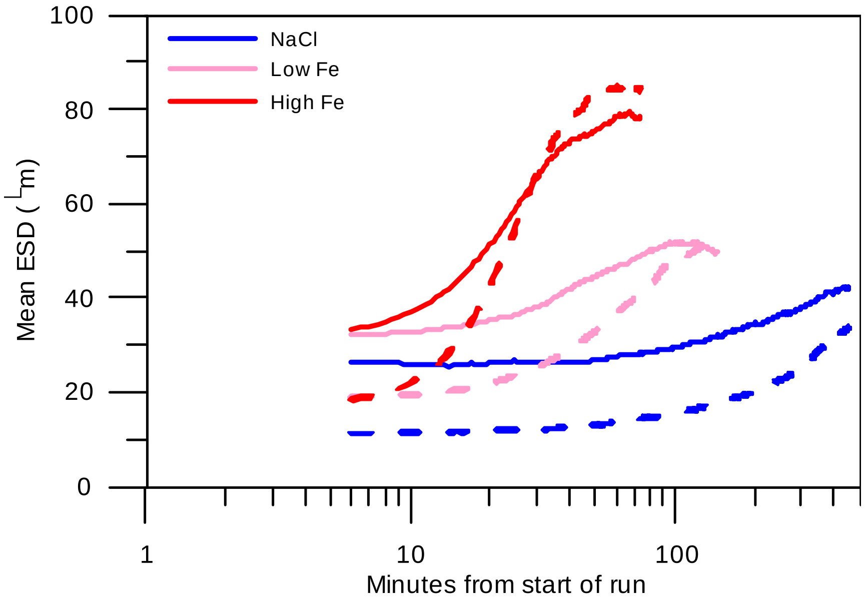

Figure S8. Treatment-specific changes in mean ESD over time. Solid lines are Pcam data, and dashed lines are LISST data.

Treatment	r-value, All data
**High Fe**	0.94
**Low Fe**	0.91
**NaCl**	0.97

Table S4. Correlation coefficients (r-values) between LISST and Pcam measurements for the three treatments shown in Fig. S6. For all values, $p < 0.0001$.

Because the LISST has a lower size limit than the Pcam, the mean ESD measured by the LISST tends to be smaller in situations where there are many single fine-grained particles, e.g., at the start of the flocculation experiments (see Figure S). When both systems are measuring floc sizes, the PSD from the LISST tends to be better sorted and closer to having a log-normal distribution. This is probably caused by the scattering theory used to invert the scattering signal of the LISST to a PSD, meaning that the data are smoothed towards a log-normal distribution by the LISST inversions. The procedure for calculating the ESD from the Pcam data does not rely on any inversions or assumptions about distribution shape; thus, we believe the Pcam data to be closer to reality. Despite these differences, highly significant correlations are observed in all experiments.
